# Heart Rate Recovery in Asymptomatic Patients with Chagas Disease

**DOI:** 10.1371/journal.pone.0100753

**Published:** 2014-06-30

**Authors:** Maria Clara Noman de Alencar, Manoel Otávio da Costa Rocha, Márcia Maria de Oliveira Lima, Henrique Silveira Costa, Giovane Rodrigo Sousa, Renata de Carvalho Bicalho Carneiro, Guilherme Canabrava Rodrigues Silva, Fernando Vieira Brandão, Lucas Jordan Kreuser, Antonio Luiz Pinho Ribeiro, Maria Carmo Pereira Nunes

**Affiliations:** 1 Post-Graduate Program in Infectious Diseases and Tropical Medicine, School of Medicine, Universidade Federal de Minas Gerais, Belo Horizonte, MG, Brazil; 2 School of Medicine, Universidade Federal de Minas Gerais, Belo Horizonte, MG, Brazil; 3 University of Minnesota Medical School, Minneapolis, Minnesota, United States of America; Johns Hopkins University SOM, United States of America

## Abstract

**Background:**

Chagas disease patients with right bundle-branch block (RBBB) have diverse clinical presentation and prognosis, depending on left ventricular (LV) function. Autonomic disorder can be an early marker of heart involvement. The heart rate recovery (HRR) after exercise may identify autonomic dysfunction, with impact on therapeutic strategies. This study was designed to assess the HRR after symptom-limited exercise testing in asymptomatic Chagas disease patients with RBBB without ventricular dysfunction compared to patients with indeterminate form of Chagas disease and healthy controls.

**Methods:**

One hundred and forty-nine subjects divided into 3 groups were included. A control group was comprised of healthy individuals; group 1 included patients in the indeterminate form of Chagas disease; and group 2 included patients with complete RBBB with or without left anterior hemiblock, and normal ventricular systolic function. A symptom-limited exercise test was performed and heart rate (HR) response to exercise was assessed. HRR was defined as the difference between HR at peak exercise and 1 min following test termination.

**Results:**

There were no differences in heart-rate profile during exercise between healthy individuals and patients in indeterminate form, whereas patients with RBBB had more prevalence of chronotropic incompetence, lower exercise capacity and lower HRR compared with patients in indeterminate form and controls. A delayed decrease in the HR after exercise was found in 17 patients (15%), 9% in indeterminate form and 24% with RBBB, associated with older age, worse functional capacity, impaired chronotropic response, and ventricular arrhythmias during both exercise and recovery. By multivariable analysis, the independent predictors of a delayed decrease in the HRR were age (odds ratio [OR] 1.11; 95% confidence interval [CI] 1.03 to 1.21; p = 0.010) and presence of RBBB (OR 3.97; 95% CI 1.05 to 15.01; p = 0.042).

**Conclusions:**

A small proportion (15%) of asymptomatic Chagas patients had attenuated HRR after exercise, being more prevalent in patients with RBBB compared with patients in indeterminate form and controls.

## Introduction

Chagas disease remains a serious health problem in Latin America, being a leading cause of heart disease [1]–[3]. Although symptoms of chronic heart disease appear several years after initial infection, progressive myocardial damage occurs throughout early stages [4], [5]. The development of electrocardiographic (ECG) abnormalities implies disease progression and usually precedes the appearance of symptoms [6]–[8]. In particular, patients with conduction system abnormalities expressed by right bundle-branch block (RBBB) are at risk for developing progressive myocarditis [6]. Nevertheless, there is a wide spectrum of Chagas disease patients with RBBB from asymptomatic with normal ventricular function to severe heart failure, and the prognosis depends mainly on left ventricular (LV) function [4], [9], [10]. Moreover, the subset of Chagas disease patients with RBBB and normal ventricular function encompasses a heterogeneous population with variable response to physical exercise in terms of functional performance and exercise-induced arrhythmias.

Autonomic disorder is an early alteration in the course of heart impairment in Chagas disease, which has also been implicated in the pathophysiology of the ventricular dysfunction [11]–[14]. A decline in heart rate (HR) after exercise is an index of autonomic activity that reflects parasympathetic reactivation [15], [16]. An attenuated heart rate recovery (HRR) after exercise has been shown to be associated with impaired functional capacity and poor prognosis among people with and without cardiovascular disease [17]–[21].

We believe that HRR might be a useful tool for autonomic nervous system evaluation in the setting of Chagas disease. Therefore, the present study was designed to assess the HRR after maximal symptom-limited exercise test in asymptomatic Chagas disease patients with RBBB without ventricular dysfunction compared to patients in indeterminate form and healthy controls.

## Methods

### Study Population

Patients with the diagnosis of chronic Chagas disease, evaluated at our institution from October 2010 to April 2012, were initially considered for the study. Diagnosis of Chagas disease required at least two positive serologic tests for antibodies against *T. cruzi.* The study was approved by the Research Ethics Committee of the Federal University of Minas Gerais, Brazil, and an informed written consent was obtained from all subjects.

To be included in the study, patients should be asymptomatic, in sinus rhythm, and with normal left ventricular systolic function by echocardiography. Patients who were unable to perform an exercise test or had prior diagnosis of heart disease from other etiology were excluded. In addition, patients with other systemic diseases that can be associated with autonomic dysfunction, especially hypertension and diabetes, were not included.

### Electrocardiography evaluation

ECG was analyzed by an experienced cardiologist who was blinded to the serological tests for Chagas disease following the recommendations for the standardization and interpretation of the electrocardiogram [22].

Diagnostic criteria for complete RBBB were QRS duration ≥120 ms, with prominent and notched R waves (rsr', rsR', or rSR' patterns) in right precordial leads (V_1_ and V_2_) and wide S waves that are longer in duration than the preceding R wave in left precordial leads (V_5_ and V_6_) with normal QRS axis (within −30° and 90°). Left anterior fascicular block (LAFB) was considered to be associated with RBBB when QRS axis was between −45° and −90° with qR pattern in lead aVL.

**Figure 1:**
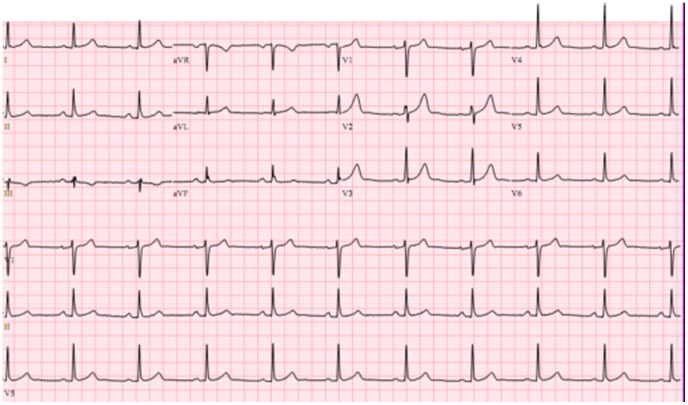
A normal ECG in a patient in the indeterminate form of Chagas disease.

Patients meeting the inclusion criteria were further categorized into 2 groups according to ECG evaluation. Group 1 included patients in the indeterminate form of Chagas disease defined as those with positive serological tests with a normal chest radiograph and ECG [5], [8] (Figure 1). In this group, patients with any minor ECG abnormalities including ST-T changes were not included.

**Figure 2:**
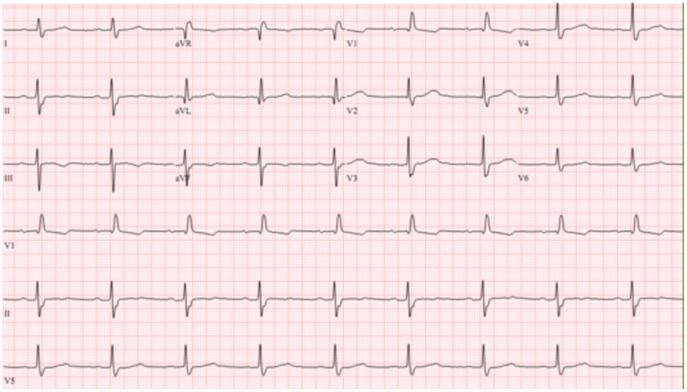
An ECG showing complete right bundle-branch block associated with left anterior hemiblock.

**Figure 3:**
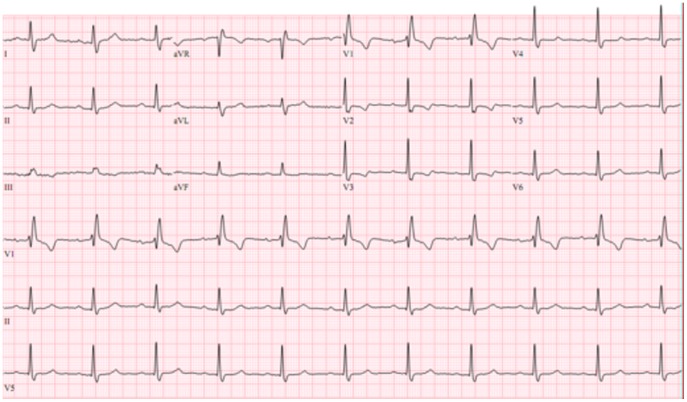
An ECG showing isolated right bundle-branch block.

Group 2 included patients with complete RBBB with or without LAFB, and normal left ventricular ejection fraction (Figures 2 and 3).

In addition, a control group comprised of healthy individuals referred for cardiovascular evaluation with normal exams was also included.

### Exercise testing protocol

A symptom-limited exercise was performed on a treadmill (Digistress Pulsar, Micromed, Brazil) using a standard Bruce protocol [23]. A physician who was unaware of the echocardiogram results was present during all of the studies to encourage maximal exertion. A 12-lead ECG was continuously monitored and recorded in each minute, and cuff blood pressure was recorded manually at rest, during the last 30 seconds of each stage and during the 6-min recovery period. After achieving maximal workload, all patients spent 1 minute in a cool-down period at a speed of 2.4 km per hour and a grade of 2.5 percent [24]. After 1-min, all of them completed recovery phase in supine position.

Heart rate was measured at rest, peak exercise and at one minute recovery. Heart rate recovery (HRR) was defined as the difference between peak HR and HR registered at the end of active recovery. An abnormal HRR at minute 1 of recovery was defined as a change from the peak HR of 12 beats or less.

Chronotropic incompetence (CI) was defined accordingly the following criteria: (1) failure to achieve 85% of age-predicted maximal heart rate (220-age) [25] and/or (2) failure to attain 80% of HR reserve (HR reserve  =  age-predicted maximal heart rate – resting HR) [26]. The proportion of heart rate reserve achieved have been termed chronotropic index, considered abnormal <80% [26], [27].

### Echocardiographic Evaluation

A standard transthoracic two-dimensional (2D) echocardiogram was performed according to recommendations of the American Society of Echocardiography using a commercially available echocardiograph (GE Vivid 7, Horten, Norway). LV ejection fraction was calculated according to the modified Simpson's rule [28].

Diastolic function was assessed by pulsed-wave Doppler examination of mitral, and tissue Doppler imaging [28], [29]. RV function was quantitatively assessed using the peak systolic tissue Doppler velocity acquired at the basal RV free wall [30].

### Statistics analysis

Normally distributed variables are reported as mean ±_SD and compared with analysis of variance (ANOVA). Data that were not normally distributed are reported as median with interquartile range and compared with Mann Whitney test. Categorical variables are reported as number and percentage and compared with chi-square test.

A HRR ≤12 beats at 1 min post-exercise was defined as an abnormal response. Logistic regression analysis was performed to determine characteristics that were independently associated with abnormal HRR. Variables that were found to be significantly associated with abnormal HRR in univariable analysis or clinically relevant were included in the multivariable logistic regression analysis. To avoid colinearity among a subset of highly similar variables, only the clinically important variables were chosen to enter into the model. SPSS software (version 19.0) was used for statistical analysis.

## Results

From 134 Chagas disease patients initially referred to the study, 23 patients (17%) were excluded: 15 patients in the indeterminate form and 8 with RBBB. Of the 15 patients excluded in the indeterminate form, 9 patients had hypertension, 4 patients had ST-T abnormalities, and 2 patients were found to have mild left ventricular enlargement on echocardiography examination. Of the 54 patients with RBBB, 5 had hypertension or diabetes, 2 had complete atrioventricular block, and one patient was unable to complete the exercise test. Therefore, a total of 111 Chagas disease patients and 38 controls who met all inclusion criteria were included.

The baseline characteristics of the patients and controls are shown in Table 1. All patients were asymptomatic, in NYHA functional class I, and 55 patients (50%) were male. Regarding medication, 9 patients (8%) were taking amiodarone (3 of whom were also taking beta-blockers). As expected, the mean QRS duration was 82.3±2.7 ms in patients in the indeterminate form compared to 136.3±15.8 ms in patients with RBBB.

**Table 1:** Characteristics of the Patients at rest.

Variable	Controls (n = 38)	Group 1 (n = 65)	Group 2 (n = 46)	p Value
Age (years)	44.1±9.2	46.9±7.9	50.9±9.9	0.001*
Male (n/%)	22 (58)	30 (46)	25 (54)	0.469
BSA (m^2^)	1.79±0.2	1.75±0.1	1.75±0.2	0.476
SBP (mmHg)	125.8±14.7	120.6±13.2	122.4±8.3	0.127
DBP (mmHg)	86.3±8.5	84.5±7.1	80.8±6.2	0.002*
LVDd (mm)	47.2±5.6	48.2±4.6	46.6±3.2	0.171
LVSd (mm)	28.6±4.2	29.8±3.9	30.1±3.1	0.209
LVEF (%)	68 [66/73]	69 [64/72]	64 [61/67]	<0.001*
LA dimension (mm)	34 [30/36]	35 [31/37]	36 [34/38]	0.009*
E wave (cm/s)	79.6±20.0	73.7±17.6	78.6±14.6	0.192
A wave (cm/s)	53 [42/69]	55 [46/64]	75 [52/85]	<0.001*
DT (ms)	185 [171/214]	192 [171/208]	233 [215/273]	<0.001*
E/e' ratio	4.1±1.4	4.4±1.1	4.0±0.9	0.158
SPAP (mmHg)	20.7±3.5	20.3±4.2	24.8±7.4	0.398
RV systolic velocity† (cm/s)	11.3±1.4	11.6±1.5	11.9±1.7	0.202

Data are expressed as mean value ± SD or median [interquartile range] or), or absolute numbers (percentage).

*p<0.05 for comparison between controls and group 1 versus group 2.

† Maximal systolic velocity at the tricuspid annulus by tissue Doppler velocity

A =  late transmitral flow velocity; BSA  =  body surface area; DBP  =  diastolic blood pressure; DT  =  deceleration time; E =  early diastolic transmitral flow velocity; E/e'  =  ratio of the early diastolic transmitral flow velocity to early diastolic mitral annular velocity; LA  =  left atrium; LVDd  =  left ventricular end-diastolic diameter; LVEF  =  left ventricular ejection fraction; LVSd  =  left ventricular end-systolic diameter; SBP  =  systolic blood pressure, SPAP  =  systolic arterial pulmonary pressure.

Of the patients with RBBB, 26 patients had RBBB associated with left anterior fascicular block (56.5%), and 20 patients (43.5%) had isolated RBBB. Others conduction system abnormalities detected in these patients were first degree atrioventricular block in 7 patients (15.2%); Mobitz type I second degree atrioventricular block in 3 patients (6.5%). Minor ECG abnormalities including sinus bradycardia were detected in 19 patients (41.3%).

Control subjects and patients in the indeterminate form were younger than the patients with RBBB. Similarly, there were no difference in echocardiographic parameters between controls and patients in the indeterminate form (group 2) whereas patients in group 3 had lower LV ejection fraction, larger left atrial dimension, and impairment of diastolic dysfunction compared to other groups. Wall motion abnormalities were found in 11 patients: 6 patients in the indeterminate form (9%) and 5 with RBBB (11%), mainly in the apical, basal inferior and inferolateral walls.

### Exercise testing

All patients completed the study protocol. No patient had angina during the exercise test or significant ischemic ST abnormalities in the electrocardiogram.

**Figure 4:**
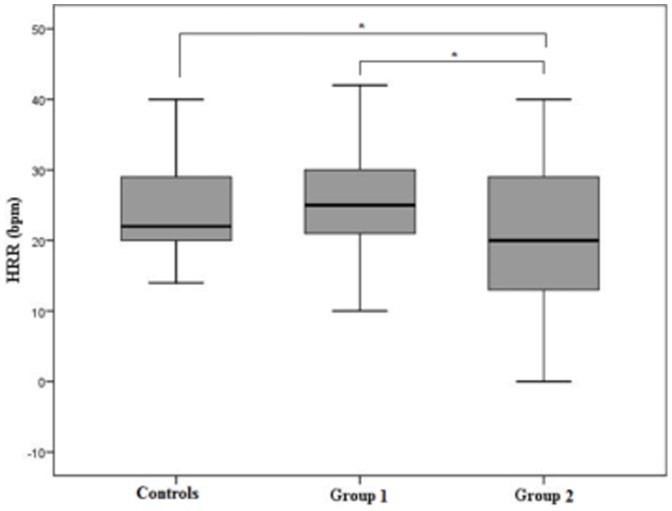
Heart rate response after exercise among the groups: healthy controls; group 1  =  patients in the indeterminate form of Chagas disease; group 2  =  patients with complete right bundle-branch block (RBBB) with or without left anterior hemiblock, and normal ventricular systolic function. Patients in group 2 had lower HRR compared to other groups. *p<0.05

Exercise test responses according to the groups are shown in Table 2. There were no differences between controls and patients in group 1. On the other hand, patients in group 2 had more prevalence of chronotropic incompetence, lower exercise capacity and lower HRR (Figure 4) compared with controls and patients in group 1.

**Table 2:** Exercise test responses according to the groups.

Variable	Controls (n = 38)	Group 1 (n = 65)	Group 2 (n = 46)	p Value
Resting HR (beats/min)	75.1±9.8	74.2±12.7	74.5±10.7	0.971
Peak HR (beats/min)	176 [166/187]	176 [165/184]	156 [136/168]	<0.001*
Peak HR (% predicted)	100 [97/103]	100 [94/107]	92 [81/99]	<0.001*
HRR (beats/min)	24.0±6.7	25.0±8.9	20.2±11.7	0.029*
HRR ≤12 bpm	0	6 (9)	11 (25)	0.001* †
Peak VO_2_ (ml.Kg^−1^.min^−1^)	40.4±7.7	40.4±10.4	35.5±10.5	0.024*
METs	11.5±2.2	11.5±3.1	10.1±3.0	0.017*
Chronotropic index	0.99±0.1	0.98±0.2	0.82±0.2	<0.001*
Chronotropic index (<80%)	1 (3)	4 (6)	20 (43)	<0.001*
Peak exercise HR (<85% of the age-predicted HR	1 (3)	3 (5)	18 (39)	<0.001*

Data are expressed as mean value ± SD, number (percentage) of patients or median [interquartile range].

*p<0.05 for comparison between controls and group 1 versus group 2.

† p = 0.058 for comparison between controls and group 1.

HR  =  heart rate; HRR  =  heart rate recovery; MET  =  metabolic equivalents

**Figure 5:**
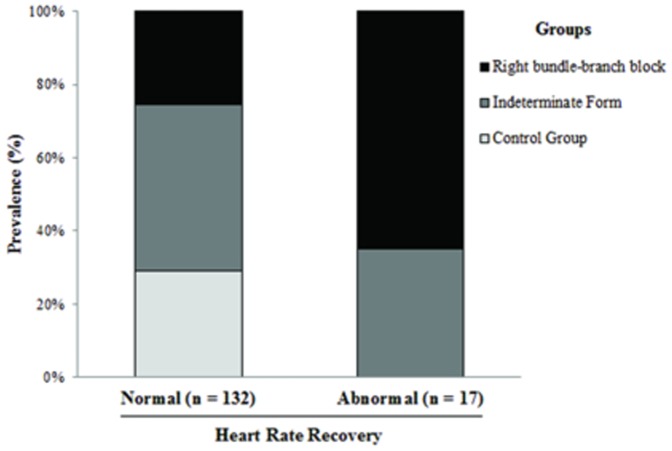
Proportion of abnormal HRR according to the groups. Abnormal HRR was gradually more prevalent across groups from controls, patients with the indeterminate form to patients with RBBB.

The median value for heart-rate recovery was 24 beats per minute, with a range from the 25th to the 75th percentile of 19 to 29 beats per minute. An abnormal value for heart-rate recovery was found in 17 patients (15%), 9% in indeterminate form and 24% with RBBB. No subjects in control group had HRR ≤12 beats/min (Figure 5).

Characteristics of the study participants according to HRR are summarized in Table 3.

**Table 3:** Predictors of abnormal response of the HRR in the study population.

Variable	Normal (HRR>12bpm)	Abnormal (HRR≤12bpm)	OR (95%CI)	p Value
Age (years)	47.1±8.2	57.3±9.3	1.16 (1.08–1.25)	<0.001
Use of amiodarone	5 (5)	4 (24)	6.4 (1.59–25.48)	0.026
LVEF (%)	66.9±5.2	65.8±3.8	0.91 (0.82–1.02)	0.282
RBBB (n/%)	33 (35)	11 (65)	5.39 (1.85–5.72)	0.002
E (cm/s)	76.4±16.7	71.9±15.3	0.99 (0.99–1.00)	0.180
A (cm/s)	59.9±18.8	78.2±22.3	1.01 (1.00–1.01)	<0.001
DT (ms)	209.1±42.6	240.8±61.5	1.01 (1.00–1.02)	0.011
E/e' ratio	4.2±1.1	4.3±0.9	1.12 (0.69–1.83)	0.744
Resting HR (beats/min)	70.5±10.1	70.7±13.9	1.01 (0.97–1.06)	0.961
Peak HR (beats/min)	168.7±19.6	146.1±27.7	0.95 (0.93–0.98)	<0.001
Peak HR (% predicted)	97.9±10.6	89.5±15.7	0.93(0.89–0.98)	0.002
Peak VO_2_ (ml.Kg^−1^.min^−1^)	39.7±10.3	33.1±9.6	0.92 (0.86–0.98)	0.016
Frequent VPB during exercise*	17 (18)	7 (41)	3.97 (1.32–1.89)	0.014
Frequent VPB during recovery*	14 (15)	8 (47)	5.55 (1.80–7.11)	0.003
Peak exercise HR (<85% of the age-predicted HR)	15 (16)	5 (29)	2.97 (0.92–9.54)	0.200
Chronotropic index (<80%)	15 (16)	7 (41)	4.94 (1.65–4.83)	0.004

Data are expressed as mean value ± SD or number (percentage) of patients.

LVEF  =  left ventricular ejection fraction; RBBB  =  right bundle branch block; E =  early diastolic transmitral flow velocity; A =  late transmitral flow velocity; DT  =  deceleration time; ms  =  milliseconds; E/e'  =  ratio of the early diastolic transmitral flow velocity to early diastolic mitral annular velocity; HR  =  heart rate; VO_2_  =  oxygen uptake; VPB  =  ventricular premature beats.

*defined as more than 7 ventricular premature beats per minute [30].

There was a weak but significant correlation between the peak HR and HRR (r = 0.2; p = 0.015). However, no correlation was found when peak heart rate was expressed as a percentage of age-predicted maximal HR.

As compared with the patients with a normal value for HRR, those with an abnormal value (≤12 beats/min) were older, had more frequent abnormal rest ECG with RBBB, lower peak HR values in percentage of predicted, lower exercise capacity, and were more likely to have diastolic dysfunction and chronotropic incompetence as assessed by chronotropic index. Additionally, these patients had more frequent ventricular ectopy during both exercise and recovery. Likewise, use of amiodarone was more frequent in those with abnormal HRR. There were no differences in the LV ejection fraction or LV filling pressures between patients with normal and abnormal HRR (Table 3).

The variables included in the final multivariable model were age, use of amiodarone, resting HR, peak VO_2_, left ventricular ejection fraction, E/e' ratio, and the presence of RBBB. The variables that remained independently associated with a delayed decrease in the HRR were age (OR 1.11; 95% CI 1.03 to 1.21; p = 0.010) and presence of RBBB (OR 3.97; 95% CI 1.05 to 15.01; p = 0.042).

## Discussion

The present study addresses the value of recovery of the HR immediately after maximal symptom-limited exercise test as a marker of autonomic dysfunction in asymptomatic patients with Chagas disease. The principal findings of this study were (1) patients classified as in indeterminate form of the disease had heart-rate responses to exercise similar to healthy individuals; (2) abnormal HRR was detected in 17 patients (15%), 9% in indeterminate form and 24% with RBBB; and (3) a delayed decrease in the HR after exercise was associated with older age, worse functional capacity, impaired chronotropic response, and ventricular arrhythmias. These findings support the concept that abnormalities in autonomic balance may precede manifestations of cardiovascular disease in Chagas disease.

To the best of our knowledge, this is the first study to demonstrate abnormal HRR in asymptomatic Chagas disease patients. Previous study has shown that ECG during the early asymptomatic stage of infection is able to identify patients at high risk for developing progressive myocarditis. [6] In contrast, patients with normal ECG have a good prognosis with mortality rates similar to those of non-Chagas persons [6], [31]. RBBB is a typical ECG abnormality of Chagas disease, unequivocally related to the cardiac involvement by the disease. However, in patients with RBBB the response to physical exercise is variable in terms of functional performance and exercise-induced arrhythmias. Therefore, even in RBBB Chagas disease patients with normal ventricular systolic function, physical effort can trigger arrhythmias, which might be associated with the changes in autonomic function during exercise. The HRR may provide further information regarding the risk for arrhythmias that occur in the setting of exercise or recovery. Our findings demonstrate that abnormal HRR was gradually more prevalent compared patients in the indeterminate form to patients with RBBB. This subset of chagasic patients who had abnormal HRR has a good prognosis in terms of symptoms and ventricular function, but they may be at risk for developing arrhythmias during exercise.

### Autonomic Abnormalities in Chagas disease

The autonomic nervous system of patients with Chagas disease has been extensively studied [11], [32], [33]. Previous studies demonstrated destruction of the parasympathetic ganglions cells in the heart due to the inflammatory process [32] and the presence of auto-antibodies with cholinergic antagonistic activity [13]. Patients presenting with chronotropic insufficiency in exercise test had both higher levels of anti-cholinergic autoantibodies [34] and reduced heart rate variability vagal indexes [35].

Abnormalities of autonomic HR control have been identified early in the course of the disease [11], [12], [33]. However, the implications of autonomic dysfunction in the pathogenesis of the chronic myocarditis, as well as its value in predicting adverse outcome remain a matter of debate. The destruction of the parasympathetic innervations may induce an increased sympathetic tone with a direct effect in the genesis of ventricular arrhythmias and sudden death [36]. In our study, chronotropic incompetence was found in asymptomatic patients with RBBB, whereas those patients in indeterminate form were similar to the controls. In agreement with previous investigation [35], our patients with the early manifestations of Chagas heart disease without ventricular dysfunction had impaired chronotropic response as defined by either a low percent of age-predicted HR achieved or a low percent HR reserve.

Azarbal et al [26] demonstrated that percent HR reserve was superior to the traditional percent of age-predicted HR in predicting cardiac death. Additionally, in that study percent HR reserve captured greater number of at-risk subjects that manifested a normal chronotropic response as assessed by the percent of age-predicted HR. In our study failure to reach 85% of the age-predicted maximum HR was similar between patients with normal and abnormal HRR. However, when the percent HR reserve was used as the criterion for CI, the prevalence of CI was greater in the patients with abnormal HRR compared to those with normal HRR. Therefore, the HR reserve approach has been used as the standard to assess the adequacy of HR response to exercise [37].

### Heart rate response after exercise

In the present study we assessed the capacity of the HR to decelerate after exercise as an additional measurement of the autonomic nervous system function. The rapid decline in HR after exercise expresses sympathetic withdrawal and increased parasympathetic tone to the sino-atrial node. A low HRR value has been associated with increased all-cause mortality risk in a variety of asymptomatic and diseased populations [38], even after adjustment for severity of cardiovascular disease, LV function, and exercise capacity [39]. In the present study, the patients who had an abnormal value of HRR also presented with other markers of unfavorable prognosis, which includes worse exercise capacity, chronotropic incompetence, and ventricular arrhythmias. In particular, these patients had frequent ventricular ectopy either during exercise or recovery. In a large cohort of 29,244 patients from Cleveland Clinic, only the occurrence of frequent ventricular ectopy during recovery was independent predictor of death [40] which was superior in outcome prediction than ventricular ectopy occurring only during exercise. Similar to what occurs with ventricular ectopy, recovery of the HR immediately after exercise is a function of parasympathetic function, and might have a potential prognostic role in the setting of Chagas disease. It is important to note that this additional clinical information is derived at no additional cost or effort, and HRR is routinely measured during standard exercise testing.

Recently, Cahalin et al. [41] analyzing a cohort of heart failure patients demonstrated that HRR after the 6 min walk test (6MWT) is a powerful predictor of cardiac events that performs better than distance ambulated as the reference measure. Importantly, the predictive accuracy of HRR after the 6MWT is comparable with HRR after maximal exertion during cardiopulmonary exercise testing. In view of these findings, our study is particularity relevant since the HR response after exercise can be also evaluated at sub-maximal testing, especially for patients with Chagas disease who are unable to undergo a maximal symptom-limited evaluation. Additionally, even in those patients who had chronotropic incompetence, the HRR can be evaluated as an additional marker of autonomic dysfunction. Therefore, as the prognostic information of HRR is not only related to exercise performance or maximal HR achieved, this simple measure could be employed in clinical practice and is potentially applicable to the broad spectrum of Chagas disease patients.

The influence of the achieved maximal HR on HRR is not well understood. Myers et al. [42] found that both chronotropic incompetence and abnormal HRR are independent predictors for cardiovascular mortality and they may act as independent clinical predictors. Zaim et al. [43] found a direct correlation between the peak HR obtained during a symptom-limited exercise test and the subsequent HRR. In our study, although a weak correlation between maximal HR achieved and HRR was found, there was no correlation of percent-predicted maximal HR achieved and HRR. Further study and understanding of physiological mechanisms that control and possibly link the processes of maximal HR achieved and HRR are needed.

### Study limitations

Heart rate variability, which represents the autonomic balance between the sympathetic and parasympathetic pathways acting on the intrinsic rhythm of the heart, was not assessed in this study. In addition, we did not exclude patients who were on medications that can alter the HR response to exercise. However, when we re-analyzed the data excluding 9 patients (8%) using amiodarone, the results did not change. Similarly, Cole et al [24] did not find associations between the use of beta-blockers or calcium-channel blockers and an abnormal value for HRR.

### Clinical implications

Autonomic disorder is an early alteration in the course of heart impairment in Chagas disease, which has been implicated in the pathophysiology of the ventricular dysfunction. HRR proved to be a simple, reliable and noninvasive parameter to assess the autonomic balance during exercise, useful in detecting early changes in Chagas heart disease.

The results of the present study have great medical and social importance because Chagas disease patients often come from impoverished, poorly-educated backgrounds and work in physically demanding jobs including farming, construction, and housekeeping. Abnormal HRR during exercise may be helpful to clinical-decision making regarding patients management and labor counseling. As physical effort can trigger arrhythmias, assessment of HRR seems to offer important information by identifying patients who are at high risk of exercise-induced arrhythmias.

## Conclusions

This study showed that a small proportion (15%) of asymptomatic Chagas patients had abnormal heart-rate responses to exercise, especially attenuated HRR after exercise, being more prevalent in patients with RBBB compared with patients in indeterminate form and controls. HRR assessment can identify autonomic dysfunction and could be helpful for clinical risk stratification in the setting of Chagas disease.
